# Effects of empagliflozin in patients with chronic kidney disease from Japan: exploratory analyses from EMPA–KIDNEY

**DOI:** 10.1007/s10157-024-02489-4

**Published:** 2024-04-20

**Authors:** Masaomi Nangaku, William G. Herrington, Shinya Goto, Shoichi Maruyama, Naoki Kashihara, Kohjiro Ueki, Jun Wada, Hirotaka Watada, Eitaro Nakashima, Ryonfa Lee, Dan Massey, Kaitlin J. Mayne, Aiko Tomita, Richard Haynes, Sibylle J. Hauske, Takashi Kadowaki

**Affiliations:** 1 Division of Nephrology and Endocrinology, The University of Tokyo School of Medicine, Tokyo, 113-8655 Japan; 2 Medical Research Council Population Health Research Unit, Clinical Trial Service Unit and Epidemiological Studies Unit, Nuffield Department of Population Health, University of Oxford, Oxford, UK; 3 Tokai University School of Medicine, Isehara, Japan; 4 Department of Nephrology, Nagoya University Graduate School of Medicine, Nagoya, Japan; 5 Kawasaki Medical School, Kurashiki, Japan; 6 Diabetes Research Center, Research Institute, National Center for Global Health and Medicine, Tokyo, Japan; 7 Department of Nephrology, Rheumatology, Endocrinology and Metabolism, Faculty of Medicine, Dentistry and Pharmaceutical Sciences, Okayama University, Okayama, Japan; 8 Department of Metabolism &Endocrinology, Juntendo University Graduate School of Medicine, Tokyo, Japan; 9 Department of Diabetes and Endocrinology, Chubu Rosai Hospital, Nagoya, Japan; 10 Elderbrook Solutions GmbH On Behalf of Boehringer Ingelheim Pharma GmbH & Co.KG, Biberach, Germany; 11 Boehringer Ingelheim International GmbH, Ingelheim, Germany; 12 Vth Department of Medicine, University Medical Center Mannheim, University of Heidelberg, Mannheim, Germany; 13 The University of Tokyo School of Medicine/Toranomon Hospital, Tokyo, Japan

**Keywords:** Sodium–glucose co-transporter-2 inhibitor, Kidney function, Cardiovascular disease, Randomised trial

## Abstract

**Background:**

EMPA–KIDNEY assessed the effects of empagliflozin 10 mg once daily vs. placebo in 6609 patients with chronic kidney disease (CKD) at risk of progression, including 612 participants from Japan.

**Methods:**

Eligibility required an estimated glomerular filtration rate (eGFR) of ≥ 20 < 45; or ≥ 45 < 90 ml/min/1.73m^2^ with a urinary albumin-to-creatinine ratio (uACR) of ≥ 200 mg/g. The primary outcome was a composite of kidney disease progression (end-stage kidney disease, a sustained eGFR decline to < 10 ml/min/1.73m^2^ or ≥ 40% from randomization, or renal death) or cardiovascular death. In post-hoc analyses, we explored the effects of empagliflozin in participants from Japan vs. non-Japan regions, including additional models assessing whether differences in treatment effects between these regions could result from differences in baseline characteristics.

**Results:**

Japanese participants had higher levels of albuminuria and eGFR than those from non-Japan regions. During a median of 2.0 year follow-up, a primary outcome occurred in 432 patients (13.1%) in the empagliflozin group and in 558 patients (16.9%) in the placebo group (hazard ratio [HR], 0.72, 95% confidence interval [95%CI] 0.64–0.82; *P* < 0.0001). Among the participants from non-Japan regions, there were 399 vs. 494 primary outcomes (0.75, 0.66–0.86), and 33 vs. 64 (0.49, 0.32–0.75; heterogeneity *p* = 0.06) in Japan. Results were similar when models explicitly considered treatment interactions with diabetes status, categories of eGFR/uACR, and recruitment in Japan (heterogeneity *p* = 0.08). Safety outcomes were broadly comparable between the two groups, and by Japanese status.

**Conclusions:**

Empagliflozin safely reduced the risk of “kidney disease progression or cardiovascular death” in patients with CKD, with consistent effects in participants from Japan.

**Supplementary Information:**

The online version contains supplementary material available at 10.1007/s10157-024-02489-4.

## Introduction

Four large-scale placebo-controlled outcome trials have studied the effects of sodium–glucose co-transporter-2 (SGLT2) inhibitors in different populations of patients with chronic kidney disease (CKD). Meta-analysis has demonstrated clear net benefits of SGLT2 inhibitors in the CKD populations studied, irrespective of the presence or absence of diabetes. Nevertheless, some at-risk populations remain understudied [1]. Japan has one of the highest prevalences of kidney failure per million population internationally [2], and has historically been under-represented in trials [3–5]. The EMPA–KIDNEY trial assessed the effects of empagliflozin 10 mg once daily in patients with CKD, and included the broadest range of patients at risk of progression of the four reported large SGLT2 inhibitor trials in CKD [6]. EMPA–KIDNEY’s streamlined design supported recruitment of 6609 participants from only 8 countries, with 612 participants from 25 clinical sites in Japan. In this report, we aimed to explore *post-hoc* comparisons of the effects of empagliflozin in participants from Japan vs. non-Japan regions (and include analyses comparing effects by the other regions).

## Methods

Details of EMPA–KIDNEY’s rationale [7], design [8], protocol, pre-specified data analysis plan, and main results have been reported previously [6]. The trial design was streamlined: extra work for collaborating doctors and hospitals was kept to a minimum, and only essential information was collected. Participant-reported information recorded by participant interview directly into bespoke computer systems and centrally measured creatinine were the main means of data collection. The trial was conducted at 241 centers. Regulatory authorities and ethics committees for each center approved the trial. Adults with a race-adjusted CKD–EPI [9] estimated glomerular filtration rate (eGFR) of ≥ 20 but < 45 ml/min/1.73m^2^ (irrespective of level of albuminuria); or an eGFR of ≥ 45 but < 90 ml/min/1.73 m^2^ with a urinary albumin-to-creatinine ratio (uACR) ≥ 200 mg/g at the screening visit were eligible. They were also required to be prescribed a clinically appropriate dose of single-agent renin–angiotensin system (RAS) inhibitor, where indicated and tolerated. Polycystic kidney disease was the only excluded primary kidney diagnosis. Additional procedures were required in Japan due to regulator requests. These included an extra scheduled visit within the first 4 weeks following randomization and reporting of all participant-reported non-serious adverse events throughout the trial.

The pre-specified primary outcome was time to first occurrence of the composite outcome of kidney disease progression or cardiovascular death. Kidney disease progression included end-stage kidney disease (ESKD), defined as commencing maintenance dialysis or receipt of a kidney transplant; a sustained decline in estimated eGFR to < 10 ml/min/1.73 m^2^; a sustained decline in eGFR of ≥ 40% from baseline; or death from kidney failure. Central laboratory serum creatinine measurements were used to calculate eGFR, with local laboratory creatinine measurement used when central results were missing. Kidney disease progression was a secondary outcome, and analyses of annual rate of change in eGFR (chronic and total slope) were tertiary outcomes, with exploratory analyses of these outcomes also pre-specified [6]. We emphasize the chronic slope over total slope (to account for the reversible acute dip in eGFR), and relative rather than absolute differences. Relative differences enable a direct test of any differences of the effects of empagliflozin between subgroups which is not usually possible with absolute differences, as the baseline absolute rate of eGFR decline in the placebo group usually varies importantly between subgroups.

Pre-specified subgrouping by region was into four groups: Europe, North America, China and Malaysia combined, and Japan. Results for all regions are provided in the supplement. This report’s focus is on the *post-hoc* subgroup comparison of Japan vs. the other non-Japan regions combined.

### Statistical analyses

All analyses were performed according to the intention-to-treat principle. A pre-specified Cox proportional hazards regression model adjusted for baseline variables specified in the minimization algorithm (age, sex, prior diabetes, eGFR, uACR, and region) was used to estimate the hazard ratios (HRs) and 95% confidence intervals (CI) for empagliflozin vs. placebo for time-to-event analyses (i.e., the primary, kidney disease progression, cardiovascular and safety outcomes) [10]. Effects of empagliflozin on annual rate of change in eGFR were assessed using pre-specified shared parameter models. Tests for heterogeneity of empagliflozin’s observed proportional effects (or differences) in subgroups were performed through the inclusion of relevant interaction terms in models [11]. Effects of empagliflozin on continuous outcomes (e.g., albuminuria) used a pre-specified mixed model repeated measures approach. Full statistical details are provided in the previously published data analysis plan and primary report [6]. In sensitivity analyses, exploratory modelling of the primary outcome accounting for the higher baseline eGFR & albuminuria in participants from Japan were performed by including pre-specified covariates plus treatment interactions with diabetes status, categories of eGFR/uACR and recruitment in Japan. The supplement includes an additional sensitivity analysis which was conducted *post-hoc* excluding data from two sites (28 randomized participants) in Japan, after regulators became aware of data integrity concerns raised by staff from a Site Management Organization. On-site and central statistical monitoring found no evidence of falsification of trial data in EMPA–KIDNEY, but these sites’ data were removed from the marketing approval application.

Analyses used SAS software, version 9.4 (SAS Institute, Cary NY, USA) and R v3.6.2.

## Results

Between May 2019 and April 2021, 6609 participants were randomized. Baseline characteristics by region are provided in Supplementary Table 1. Compared to the 5997 participants from Europe, North America, China and Malaysia combined, the 612 participants from Japan had a similar proportion without prior diabetes (53% vs. 54%) but differed with respect to several other baseline characteristics (Table 1). Participants in Japan were on average slightly older (mean age 65.3 ± 12 vs. 63.7 ± 14 years), less likely to be female (26% vs. 34%), reported less prior cardiovascular disease (15% vs. 28%), had a higher proportion of participants with glomerular disease (32% vs. 25%), a higher mean (SD) eGFR (45.2 ± 18.2 vs. 36.5 ± 13.8 mL/min/1.73m^2^), and higher median (IQR) uACR (683 [293–1514] vs. 290 [40–1030] mg/g).

**Table 1:** Baseline characteristics by Japan vs. non-Japan regions

	Japan		Non-Japan	
	Empagliflozin (*N* = 304)	Placebo (*N* = 308)	Empagliflozin (*N* = 3000)	Placebo (*N* = 2997)
DEMOGRAPHICS				
Age (years)	64.4 (12.2)	66.2 (11.8)	63.8 (14.0)	63.5 (14.0)
Female sex	86 (28.3)	75 (24.4)	1011 (33.7)	1020 (34.0)
Race				
White	0 (0.0)	0 (0.0)	1939 (64.6)	1920 (64.1)
Black	0 (0.0)	0 (0.0)	128 (4.3)	134 (4.5)
Asian	304 (100.0)	308 (100.0)	890 (29.7)	891 (29.7)
Mixed	0 (0.0)	0 (0.0)	14 (0.5)	7 (0.2)
Other	0 (0.0)	0 (0.0)	29 (1.0)	45 (1.5)
PRIOR DISEASE				
Diabetes				
Yes	135 (44.4)	155 (50.3)	1390 (46.3)	1360 (45.4)
No	169 (55.6)	153 (49.7)	1610 (53.7)	1637 (54.6)
Diabetes type				
Type 1	0 (0.0)	0 (0.0)	34 (1.1)	34 (1.1)
Type 2	127 (41.8)	148 (48.1)	1343 (44.8)	1318 (44.0)
Other/unknown	8 (2.6)	7 (2.3)	13 (0.4)	8 (0.3)
Cardiovascular disease				
Yes	40 (13.2)	50 (16.2)	821 (27.4)	854 (28.5)
No	264 (86.8)	258 (83.8)	2179 (72.6)	2143 (71.5)
Primary kidney diagnosis				
Diabetic kidney disease	94 (30.9)	109 (35.4)	938 (31.3)	916 (30.6)
Hypertension/renovascular	41 (13.5)	45 (14.6)	665 (22.2)	694 (23.2)
Glomerular	102 (33.6)	93 (30.2)	751 (25.0)	723 (24.1)
Other/unknown	67 (22.0)	61 (19.8)	646 (21.5)	664 (22.2)
CLINICAL MEASUREMENTS				
Blood pressure (mmHg)				
Systolic	133.9 (16.2)	135.9 (17.3)	136.6 (18.3)	136.7 (18.5)
Diastolic	77.4 (11.6)	78.5 (12.5)	78.1 (11.7)	78.0 (11.9)
Body mass index (kg/m^2^)	25.1 (4.1)	25.3 (4.0)	30.2 (6.7)	30.3 (6.9)
LABORATORY MEASUREMENTS				
Estimated GFR (mL/min/1.73m^2^)				
Mean (SD)	46.2 (17.9)	44.3 (18.4)	36.5 (13.8)	36.5 (13.7)
Distribution				
30	62 (20.4)	72 (23.4)	1069 (35.6)	1079 (36.0)
≥30 < 45	102 (33.6)	115 (37.3)	1365 (45.5)	1346 (44.9)
≥45	140 (46.1)	121 (39.3)	566 (18.9)	572 (19.1)
Urinary albumin-to-creatinine ratio (mg/g)				
Geometric mean (95% CI)	566 (484–661)	593 (508–693)	199 (185–213)	204 (190–220)
Median (Q1–Q3)	719 (293–1394)	602 (293–1727)	292 (39–1009)	288 (41–1041)
Distribution				
30	16 (5.3)	11 (3.6)	649 (21.6)	652 (21.8)
≥30 < 300	61 (20.1)	71 (23.1)	866 (28.9)	866 (28.9)
≥300	227 (74.7)	226 (73.4)	1485 (49.5)	1479 (49.3)
NT-proBNP (ng/L)	88 (40–208)	103 (45–215)	174 (76–442)	165 (72–437)
MEDICATIONS				
RAS inhibitor	261 (85.9)	271 (88.0)	2570 (85.7)	2526 (84.3)
Any diuretic therapy	61 (20.1)	64 (20.8)	1301 (43.4)	1389 (46.3)
Any lipid-lowering therapy	182 (59.9)	193 (62.7)	2008 (66.9)	1995 (66.6)

Data are mean (SD) or Median (Q1–Q3) for continuous data unless otherwise stated; and n (%) for categorical data

Overall (i.e., all regions combined), study treatment was generally well tolerated. At 12 months, the approximate study midpoint, overall self-reported adherence to study treatment was similar in each of the treatment groups (89.6% empagliflozin vs. 90.3% placebo; reasons for stopping have been reported elsewhere [6].) During 2.0 years of median follow-up overall, allocation to empagliflozin reduced the risk of the primary composite outcome of kidney disease progression or cardiovascular death by 28% (HR = 0.72; 95% CI 0.64–0.82; *P* < 0.0001). There were broadly similar relative effects in key subgroup analyses by diabetes status (heterogeneity *p* = 0.06), eGFR (trend *p* = 0.78), and some evidence to suggest relative benefits may be larger in patients with higher levels of uACR (trend *p* = 0.02). There were also broadly similar effects across the four pre-specified categories of region which compared participants from Europe, North America, China/Malaysia, and Japan (heterogeneity *p* = 0.06; Supplementary Fig. 1) [6]. Effects of empagliflozin on annual rate of change in eGFR by pre-specified regions suggested benefits were consistent across regions, with perhaps some evidence of larger relative reductions in chronic slope in North America (Supplementary Fig. 2).

**Fig. 1:**
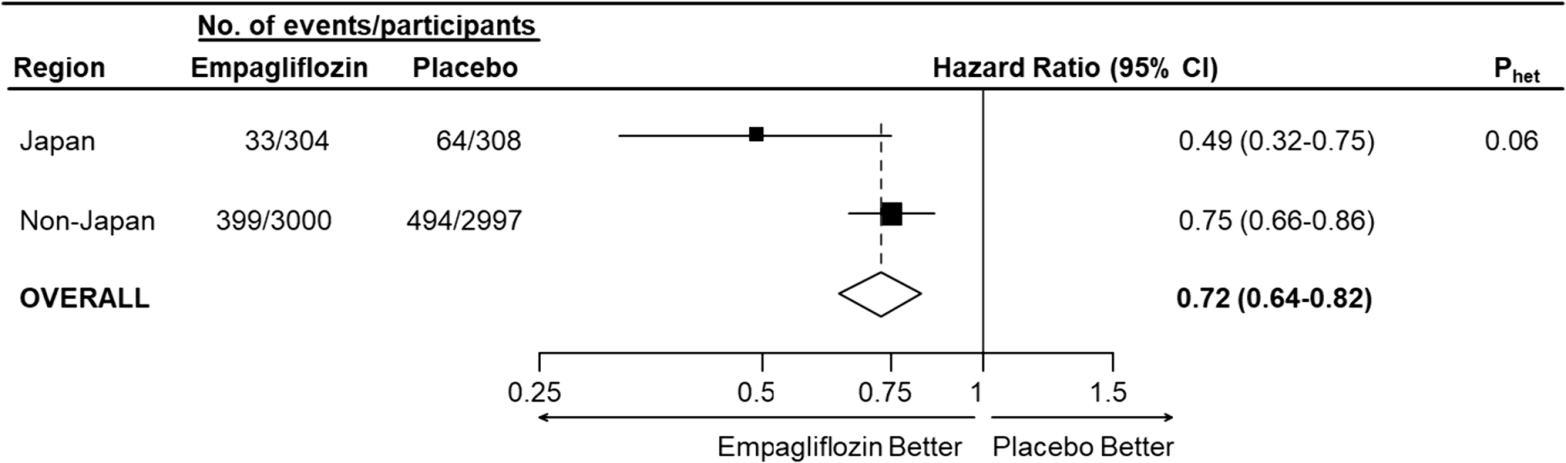
Effect of empagliflozin on primary composite outcome of progression of kidney disease or death from cardiovascular causes, by Japan vs. non-Japan regions (post-hoc exploratory analyses). Footnote: Hazard ratios and confidence intervals were estimated from Cox models, with pre-specified adjustment for age, sex, history of diabetes, estimated glomerular filtration rate (eGFR), urinary albumin-to-creatinine ratio (uACR), and region. The heterogeneity P value (P_het_) is calculated from a relevant interaction term in the pre-specified Cox model. Results were robust in a sensitivity analysis following inclusion of pre-specified covariates plus treatment interactions with diabetes status, categories of eGFR/uACR & recruitment in Japan (exploratory model P_het_ = 0.08). These non-significant heterogeneity p values suggest the best estimate of the effect of empagliflozin on the primary outcome in the two subgroups is the overall result (i.e., there is no statistical evidence for a difference between the subgroup-specific HRs for Japan vs. non-Japan regions)

Study treatment was also well tolerated in Japan. At 12 months, self-reported adherence was 91.4% for empagliflozin vs. 95.4% for placebo. Median follow-up was 1.95 years in non-Japan regions vs. 2.19 years in Japan, respectively. Among the participants from non-Japan regions, there were 399 vs. 494 primary outcomes (0.75, 0.66–0.86), whilst in Japan there were 33 vs. 64 (0.49, 0.32–0.75; heterogeneity *p* = 0.06, Fig. 1). Estimates of relative effect on the primary outcome were also similar in participants in non-Japan vs. Japan regions in sensitivity analyses which took account of differences in baseline diabetes status, eGFR, and albuminuria between participants in non-Japan and Japan regions (sensitivity analysis heterogeneity *p* = 0.08), and excluding data from the 28 participants at 2 sites excluded from the marketing authorisation application (Supplementary Table 3). Overall, the annual rate of eGFR decline in patients in the placebo group appeared generally constant (overall, and in Japan). In the empagliflozin group, the expected acute decrease in the eGFR was evident on initiation of study treatment (non-Japan −2.1 vs. Japan –2.4 mL/min/1.73 m^2^; heterogeneity *p* = 0.50), followed by slowing of the chronic rate of annual eGFR decline. The relative difference in chronic eGFR slope was a −49% (95% CI −58 to −40%) in the non-Japan regions vs. −55% in Japan (95% CI −73 to −37%; heterogeneity *p* = 0.58, Supplementary Figs. 3 and 4). Averaged over time, the difference in geometric mean uACR was −19% in the empagliflozin group compared to the placebo group (95% CI −23 to −15%), which included a −18% (95% CI −23 to −14%) difference in the non-Japan regions and a −26% (95% CI −36 to −14%) difference in Japan; heterogeneity *p* = 0.24).

The effects of empagliflozin on pre-specified secondary outcomes were broadly similar when participants were compared across the different regions (Supplementary Table 2) including in analyses comparing non-Japan vs. Japan regions (Table 2). There was a relatively low absolute risk of serious safety outcomes among the participants recruited in Japan compared to non-Japan regions, with a safety profile consistent with the overall results of the trial (Table 2).

**Table 2:** Effect of empagliflozin on primary, secondary, and safety outcomes by Japan vs. non-Japan regions (post-hoc exploratory analyses)

	Japan		Non-Japan		Overall			
	Empagliflozin (*N* = 304)	Placebo (*N* = 308)	Empagliflozin (*N* = 3000)	Placebo (*N* = 2997)	Empagliflozin (*N* = 3304)	Placebo (*N* = 3305)	Overall HR (95% CI)	Japan vs. non-Japan P_het_
	*n* (%)	*n* (%)	*n* (%)	*n* (%)	*n* (%)	*n* (%)		
Primary outcome: progression of kidney disease or death from cardiovascular causes	33 (10.9)	64 (20.8)	399 (13.3)	494 (16.5)	432 (13.1)	558 (16.9)	0.72 (0.64–0.82)	0.06
Key secondary outcomes^a^								
Hospitalization for heart failure or death from cardiovascular causes	6 (2.0)	11 (3.6)	125 (4.2)	141 (4.7)	131 (4.0)	152 (4.6)	0.84 (0.67–1.07)	0.56
Death from any cause	7 (2.3)	7 (2.3)	141 (4.7)	160 (5.3)	148 (4.5)	167 (5.1)	0.87 (0.70–1.08)	0.52
Other secondary outcomes								
Progression of kidney disease	31 (10.2)	63 (20.5)	353 (11.8)	441 (14.7)	384 (11.6)	504 (15.2)	0.71 (0.62–0.81)	0.04
Death from cardiovascular causes	3 (1.0)	1 (0.3)	56 (1.9)	68 (2.3)	59 (1.8)	69 (2.1)	0.84 (0.60–1.19)	–
End-stage kidney disease or death from cardiovascular causes^b^	10 (3.3)	16 (5.2)	153 (5.1)	201 (6.7)	163 (4.9)	217 (6.6)	0.73 (0.59–0.89)	0.88
Safety outcomes								
Serious urinary tract infection	1 (0.3)	2 (0.6)	51 (1.7)	52 (1.7)	52 (1.6)	54 (1.6)	0.94 (0.64–1.37)	0.67
Serious genital infection	0 (0.0)	0 (0.0)	1 (< 0.1)	1 (< 0.1)	1 (< 0.1)	1 (< 0.1)	–	–
Serious hyperkalemia	1 (0.3)	2 (0.6)	91 (3.0)	107 (3.6)	92 (2.8)	109 (3.3)	0.83 (0.63–1.09)	0.76
Serious acute kidney injury	1 (0.3)	4 (1.3)	106 (3.5)	131 (4.4)	107 (3.2)	135 (4.1)	0.78 (0.60–1.00)	0.35
Serious dehydration	2 (0.7)	3 (1.0)	28 (0.9)	21 (0.7)	30 (0.9)	24 (0.7)	1.25 (0.73–2.14)	0.62
Liver injury	2 (0.7)	0 (0.0)	11 (0.4)	12 (0.4)	13 (0.4)	12 (0.4)	1.09 (0.50–2.38)	0.99
Ketoacidosis^c^	0 (0.0)	0 (0.0)	6 (0.2)	1 (< 0.1)	6 (0.2)	1 (< 0.1)	–	–
Lower-limb amputation	2 (0.7)	0 (0.0)	26 (0.9)	19 (0.6)	28 (0.8)	19 (0.6)	1.43 (0.80–2.57)	0.98
Bone fracture	17 (5.6)	14 (4.5)	116 (3.9)	109 (3.6)	133 (4.0)	123 (3.7)	1.08 (0.84–1.38)	0.66
Severe hypoglycemia^d^	10 (3.3)	6 (1.9)	67 (2.2)	71 (2.4)	77 (2.3)	77 (2.3)	1.00 (0.73–1.37)	0.21
Symptomatic dehydration^e^	6 (2.0)	6 (1.9)	77 (2.6)	70 (2.3)	83 (2.5)	76 (2.3)	1.10 (0.81–1.51)	0.96

The P_het_ is the p value for heterogeneity between treatment allocation and participation in Japan vs. non-Japan regions: after considering multiplicity of testing there is no statistical evidence of a difference in the HRs between participants in Japan vs. non-Japan regions. Hazard ratios and heterogeneity p values were not calculated for outcomes with fewer than 10 events

^a^The analysis of hospitalizations for any cause (a key secondary analyses) included the first and all subsequent events so only the rates are relevant: overall, for the empagliflozin vs. placebo groups these rates were 24.8 vs. 29.2 per 100 patient-years, HR 0.86 (95% CI 0.78–0.95); Japan vs. non-Japan P_het_ = 0.17

^b^End-stage kidney disease is defined as start of maintenance dialysis or receipt of a kidney transplant

^c^Ketoacidosis occurred in one patient (in the empagliflozin group) without diabetes at baseline

^d^Defined as low blood sugar causing severe cognitive impairment which requires assistance from another person for recovery

^e^Defined as whether or not a participant has experienced symptoms they attribute to dehydration, such as feeling faint or fainting

## Discussion

In the EMPA–KIDNEY population of 6609 patients with a wide range of eGFR, levels of albuminuria, and causes of CKD, empagliflozin led to a 28% lower risk of progression of kidney disease or cardiovascular death compared with placebo, and was well-tolerated. A particular feature of the trial was the recruitment of 612 participants from Japan, a country under-represented in trials and where lifetime risk of progression to kidney failure is high compared to many other high-income countries. Between-region comparisons have more limited power, but such subgroups analyses suggested relative benefits were broadly similar in patients from Europe, North America, China/Malaysia, and Japan. Exploratory analyses comparing the participants from Japan with those from other regions were consistent with this finding: there was no strong statistical evidence of differential effects between Japan and non-Japan regions before or after adjustments which accounted for higher baseline eGFR and albuminuria in Japan. These analyses should not be interpreted as suggesting greater reduction in risk of the primary outcome in Japan however, statistical power is limited by event numbers and, based on statistical tests of heterogeneity, we conclude that the best estimate of effect on the primary outcome in participants in Japan is the overall estimate of a 28% relative risk reduction.

Previous sub-analyses of DAPA–CKD [4] and CREDENCE trial data [5] included 244 and 110 participants from Japan, respectively, necessitating participants from Japan to be grouped with participants from other local countries. Such sub-analyses found that the relative effects of SGLT2 inhibitors on key outcomes were broadly similar, irrespective of the different types of participant represented across the different regions [4, 5]. Our report’s findings are consistent with data from these two other CKD trials. Effects on kidney outcomes from all three trials have been combined in a collaborative meta-analysis [1]. We conclude that the overall relative risk reductions from that meta-analysis are currently the most reliable estimates of effects of SGLT2 inhibition in patients with CKD, irrespective of region [1].

These analyses need to be considered in the context of their *post-hoc*/exploratory nature. The key limitations of EMPA–KIDNEY were the lower-than-expected number of cardiovascular outcomes and its relatively short follow-up. This highlights the need for future nephrology trial designs to aim for larger samples sizes. Globally, complex research governance and burdensome regulation has caused increased organizational and operational complexity, contributing to an unsustainable increase in the cost of trials. Such burdens may contribute to a reluctance of clinician investigators or patients to participate in trials and limit the ability to recruit at scale [13]. EMPA–KIDNEY’s streamlined design aimed to help reverse these trends. We used a risk-based approach focusing on critical-to-quality data. Future streamlined trial designs incorporating linkage to the Japan-Chronic Kidney Disease-Database could also facilitate longer-term low-cost kidney outcome follow-up [12].

In summary, it is reasonable to conclude that EMPA–KIDNEY’s main conclusions can be extrapolated to patients with CKD in Japan and non-Japan regions, with demonstrable net absolute benefits.

## Disclosure

The analyses were performed and original full database developed and held by the Nuffield Department of Population Health at the University of Oxford and validated by the funder. The sponsor provided a grant to the University of Oxford and have members on the Steering Committee who are responsible for reviewing all trial publications. WGH accepts full responsibility for the content of the paper and the decision to submit. CTSU has a staff policy of not accepting honoraria or other payments from the pharmaceutical industry, except for the reimbursement of costs to participate in scientific meetings (www.ctsu.ox.ac.uk). EMPA–KIDNEY was mainly supported by a grant to the University of Oxford from Boehringer Ingelheim, which included funding from Eli Lilly. CTSU (authors WGH, KJM, RL, RH) received core funding to the Medical Research Council Population Health Research Unit at the University of Oxford, which is part of the Clinical Trial Service Unit and Epidemiological Studies Unit (CTSU), from the UK Medical Research Council (grant numbers MC_UU_00017/3 and MC_UU_00017/4). CTSU is also supported by Health Data Research UK and the National Institute for Health and Care Research Oxford Biomedical Research Centre. WGH was supported by a Medical Research Council Kidney Research UK Professor David Kerr Clinician Scientist Award (MR/R007764/1). MN reports research grants from Boehringer Ingelheim, Kyowa-Kirin, Daiichi-Sankyo, Astellas, Tanabe-Mitsubishi, JT, Chga, Torii, Takeda, Bayer; consulting fees from Kyowa-Kirin, Astellas, Daiichi-Sankyo, Tanabe-Mitsubishi, JT, Boehringer Ingelheim; and honoraria from Kyowa-Kirin, Astellas, Astra Zeneca, GASK, Daiichi-Sankyo, Tanabe-Mitsubishi, Chugai, Boehringer Ingelheim. SG reports consulting fees for ANTOS, Jansen Pharma, MSD and Amgen, and fees for membership of the EMPACT–MI Steering Committee from Boehringer Ingelheim. SM reports grants from Nippon Boehringer Ingelheim Co., Ltd. NK has received consulting or speaker honoraria from AstraZeneca, Kyowa Kirin, Novo Nordisk, Novartis, Astellas, Otsuka, and Novo Nordisk. JW has received speaker honoraria from Astra Zeneca, Bayer, Boehringer Ingelheim, Daiichi Sankyo, Kyowa Kirin, Novo Nordisk, and Mitsubishi Tanabe, and receives grant support from Bayer, Chugai, Kyowa Kirin, Otsuka, Shionogi, Sumitomo, and Mitsubishi Tanabe-Pharma. HW has received honoraria for lectures from Bayer Pharma Japan, Teijin Pharma Ltd, MSD, Sanofi-Aventis K.K., Novo Nordisk, Nippon Boehringer Ingelheim, Eli Lilly, Sumitomo Pharma, Mitsubishi Tanabe Pharma, Daiichi Sankyo Company, Ltd, Abbott, Kowa Co., Ltd, Taisho Pharmaceutical, and research activities for Teijin Pharma, Abbott Japan, Nippon Boehringer Ingelheim, Sumitomo Pharma, Mitsubishi Tanabe Pharma, Lifescan Japan, Sanwa Kagaku, Taisho Pharmaceutical, Takeda Pharmaceuticals, Soiken Inc. KU has received grants from Eli Lilly, Daiichi-Sankyo, Kyowa-Kirin and Abbott Japan, grants and honoraria for lectures from Novo Nordisk, Nippon Boehringer Ingelheim, MSD, Mitsubishi Tanabe Pharma, Sumitomo Pharma, Ono and Sanofi, and honoraria for lectures from Astellas and AstraZeneca. EN has received honoraria from Novo Nordisk, Sanfi K.K, Sumitomo Pharma, Abbott Japan, and Mitsubishi Tanabe Pharma. DM is a statistical consultant for Elderbrook Solutions on behalf of Boehringer Ingelheim Boehringer Ingelheim. SH is an employee of Boehringer Ingelheim. RH reports grants or contracts from Roche, GSK/Vir and Combiphar. TK reports research grants from Nippon Boehringer Ingelheim Co., Ltd, Eli Lilly Japan K.K., Kyowa Kirin Co., Ltd, MSD Corporation, Daiichi Sankyo Co., Ltd, Novo Nordisk Pharma Ltd, Sanofi K.K., Takeda Pharmaceutical Co., Ltd, Astellas Pharma Inc, Ono Pharmaceutical Co., Ltd, Mitsubishi Tanabe Pharma Corporation, Taisho Pharmaceutical Co., Ltd; and honoraria from MSD Corporation, Takeda Pharmaceutical Co., Ltd, Mitsubishi Tanabe Pharma Corporation, Astellas Pharma Inc., Teijin Pharma Ltd, Ono Pharmaceutical Co., Ltd, Astra Zeneca K.K., Sumitomo Pharma Co., Ltd, Sanofi K.K., Eli Lilly Japan K.K., Nippon Boehringer Ingelheim Co., Ltd, Novo Nordisk Pharma Ltd, Daiichi Sankyo Co., Ltd, FUJIFILM Toyama Chemical Co., Ltd, Kowa Co., Ltd, Taisho Pharmaceutical Co., Ltd.

## Rights retention statement

For the purpose of open access, the author(s) has applied a Creative Commons Attribution (CC BY) license to any Author Accepted Manuscript version arising.

### Electronic supplementary material

Below is the link to the electronic supplementary material.

Supplementary file1 (DOCX 276 KB)

## Data Availability

The complete de-identified patient data set used for presented analyses will be available in due course and the application system to apply to use data will open 6 months after publication. Departmental policy details can be found here: https://www.ndph.ox.ac.uk/data-access. At a later date, in adherence with the Boehringer Ingelheim Policy on Transparency and Publication of Clinical Study Data, scientific and medical researchers can request access to clinical study data.

## Appendix

Recruiting collaborators in Japan are listed in the Supplementary Materials. See the Appendix of Herrington et al*.* for a full list of the EMPA–KIDNEY Collaborative Group [6].

## Declarations

Regulatory authorities and ethics committees for each centre approved the trial.
