# Supplementary material for: Effects of empagliflozin in patients with chronic kidney disease from Japan: exploratory analyses from EMPA–KIDNEY
Source: Clin Exp Nephrol. 2024 Apr 20;28(6):588–95. doi: 10.1007/s10157-024-02489-4 (PMC11116192; doi:10.1007/s10157-024-02489-4)
Supplement: Supplementary file 1 — Supplementary file1 (DOCX 276 KB) [file 10157_2024_2489_MOESM1_ESM.docx]

**SUPPLEMENTARY MATERIALS**

**Effects of Empagliflozin in Patients**

**with Chronic Kidney Disease from Japan:**

**EXPLORATORY ANALYSES FROM EMPA-KIDNEY**

The EMPA-KIDNEY Collaborative Group

**Writing Committee**:

Masaomi Nangaku^1*^, William G. Herrington^2*^, Shinya Goto^3^, Shoichi Maruyama^4^, Naoki Kashihara^5^, Kohjiro Ueki^6^, Jun Wada^7^, Hirotaka Watada^8^, Eitaro Nakashima^9^, Ryonfa Lee^2^, Dan Massey^10^, Kaitlin J. Mayne^2^, Aiko Tomita^3^, Richard Haynes^2*^, Sibylle J. Hauske^11,12*^, Takashi Kadowaki^13*^

*Joint contribution.

**Correspondence**:

Professor Masaomi Nangaku,

Division of Nephrology and Endocrinology,

The University of Tokyo Graduate School of Medicine,

73-1 Hongo, Bunkyo-ku,Tokyo 113-8655, Japan.

E-mail: [mnangaku@m.u-tokyo.ac.jp](mailto:mnangaku@m.u-tokyo.ac.jp)

[www.empakidney.org](http://www.empakidney.org)

Clinical and Experimental Nephrology,

Official Publication of the Japanese Society of Nephrology

**SUPPLEMENTARY MATERIALS**

**Effects of Empagliflozin in Patients**

**with Chronic Kidney Disease from Japan:**

**EXPLORATORY ANALYSES FROM EMPA-KIDNEY**

Table of Contents

[SUPPLEMENTARY FIGURE 1: Effect OF EMPAGLIFLOZIN on Primary Composite Outcome BY REGIONS (PRE-SPECIFIED SUBGROUP) 3](#_Toc148711676)

[SUPPLEMENTARY FIGURE 2: EFFECT OF EMPAGLIFLOZIN ON TOTAL AND CHRONIC ESTIMATED GLOMERULAR FILTRATION RATE SLOPE BY REGION (PRE-SPECIFIED SUBGROUPS) 4](#_Toc148711677)

[SUPPLEMENTARY FIGURE 3: EFFECT OF EMPAGLIFLOZIN ON TOTAL AND CHRONIC ESTIMATED GLOMERULAR FILTRATION RATE SLOPE by Japan versus non-Japan RegionS (POST-HOC EXPLORATORY ANALYSES) 5](#_Toc148711678)

[SUPPLEMENTARY FIGURE 4: CHANGE FROM BASELINE IN THE ESTIMATED GLOMERULAR FILTRATION RATE SLOPE BY JAPAN VERSUS NON-JAPAN REGIONS (POST-HOC EXPLORATORY ANALYSES) 6](#_Toc148711679)

[SUPPLEMENTARY TABLE 1: BASELINE CHARACTERISTICS BY PRE-SPECIFIED REGIONS 7](#_Toc148711680)

[SUPPLEMENTARY TABLE 2: EFFECT OF EMPAGLIFLOZIN ON PRIMARY AND SECONDARY OUTCOMES BY PRE-SPECIFIED REGIONS (POST-HOC EXPLORATORY ANALYSES) 8](#_Toc148711681)

[SUPPLEMENTARY TABLE 3: SENSITIVITY ANALYSIS FOR THE PRIMARY OUTCOME EXCLUDING 28 RANDOMIZED PARTICIPANTS FROM TWO SITES IN JAPAN 9](#_Toc148711682)

[COLLABORATORS 10](#_Toc148711683)

# SUPPLEMENTARY FIGURE 1: Effect OF EMPAGLIFLOZIN on Primary Composite Outcome BY REGIONS (PRE-SPECIFIED SUBGROUP)

**
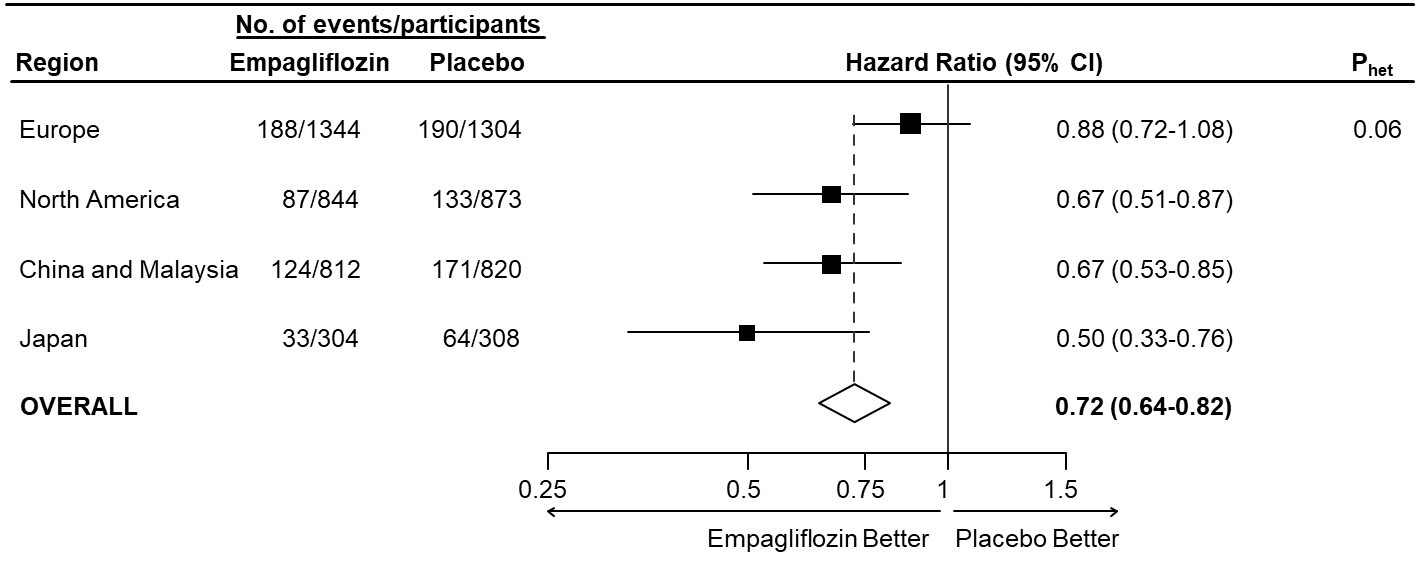
**

# SUPPLEMENTARY FIGURE 2: EFFECT OF EMPAGLIFLOZIN ON TOTAL AND CHRONIC ESTIMATED GLOMERULAR FILTRATION RATE SLOPE BY REGION (PRE-SPECIFIED SUBGROUPS)

**
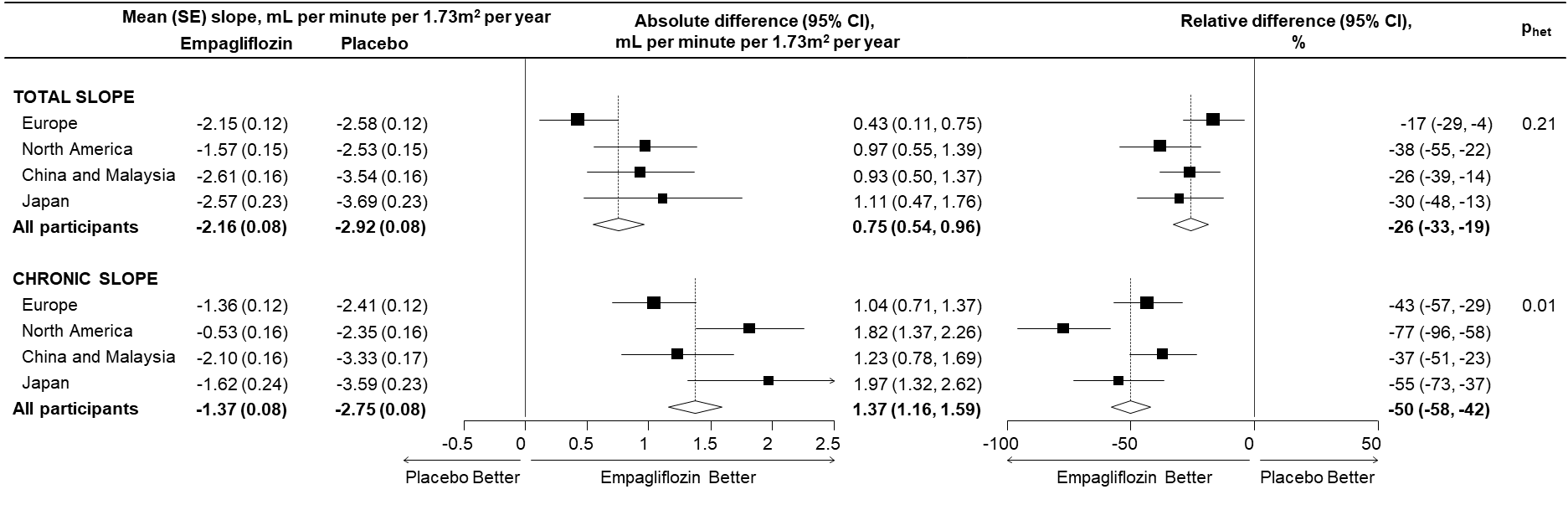
**

Mean annual rates of change in estimated GFR from baseline to the final follow-up visit (“total slopes”), and from 2 months to the final follow-up visit (“chronic slopes”) by treatment allocation were estimated using shared parameter models adjusted for age, sex, prior diabetes, urinary ACR category, and region. Models estimating chronic slope were additionally adjusted for baseline estimated GFR (as a continuous variable) and the interaction between baseline estimated GFR and follow-up time. This approach jointly models the annual rate of change in estimated GFR and the time to event for end-stage kidney disease (ESKD) or death. Analyses used all available central laboratory estimated GFR measurements prior to the development of ESKD. Relative difference is the absolute difference as a fraction of the mean slope in the placebo group, expressed as a percentage. The heterogeneity p values shown are calculated from the relative differences.

# SUPPLEMENTARY FIGURE 3: EFFECT OF EMPAGLIFLOZIN ON TOTAL AND CHRONIC ESTIMATED GLOMERULAR FILTRATION RATE SLOPE by Japan versus non-Japan RegionS (POST-HOC EXPLORATORY ANALYSES)


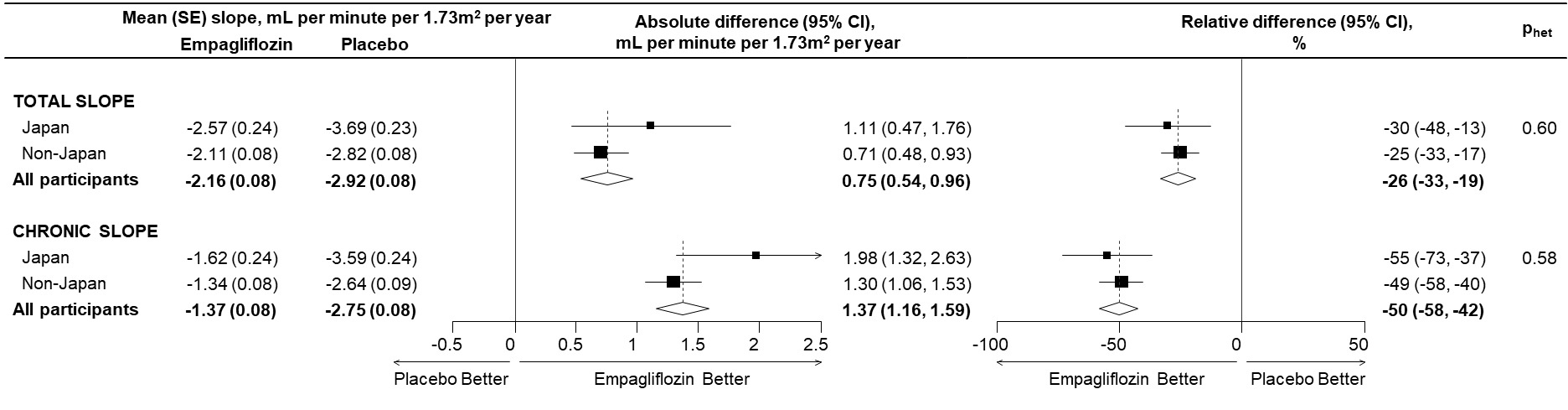


Mean annual rates of change in estimated GFR from baseline to the final follow-up visit (“total slopes”), and from 2 months to the final follow-up visit (“chronic slopes”) by treatment allocation were estimated using shared parameter models adjusted for age, sex, prior diabetes, urinary ACR category, and region. Models estimating chronic slope were additionally adjusted for baseline estimated GFR (as a continuous variable) and the interaction between baseline estimated GFR and follow-up time. This approach jointly models the annual rate of change in estimated GFR and the time to event for end-stage kidney disease (ESKD) or death. Analyses used all available central laboratory estimated GFR measurements prior to the development of ESKD. Relative difference is the absolute difference as a fraction of the mean slope in the placebo group, expressed as a percentage. The heterogeneity p values shown are calculated from the relative differences for Japan and non-Japan regions.

# SUPPLEMENTARY FIGURE 4: CHANGE FROM BASELINE IN THE ESTIMATED GLOMERULAR FILTRATION RATE SLOPE BY JAPAN VERSUS NON-JAPAN REGIONS (POST-HOC EXPLORATORY ANALYSES)


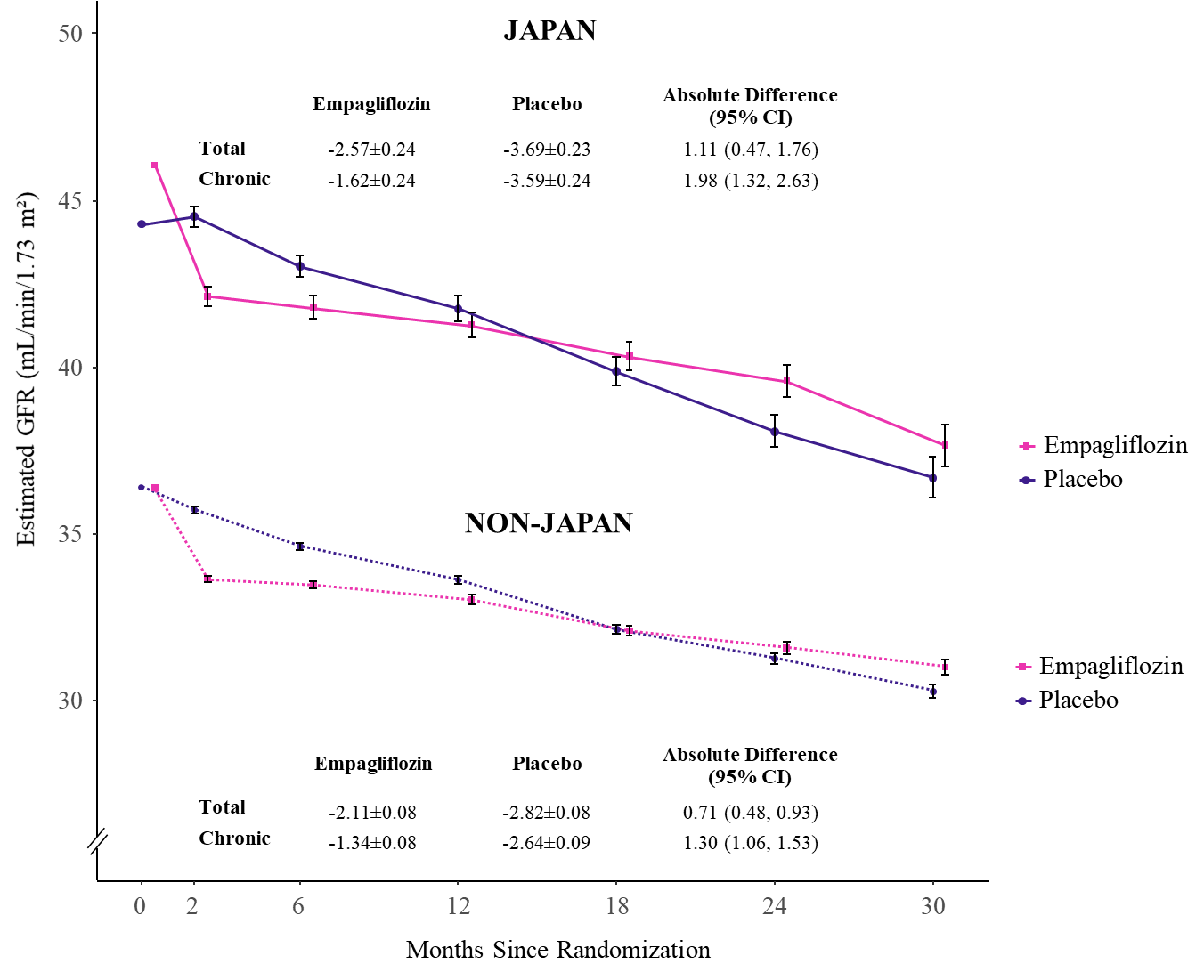


The values shown as “Total” represent the mean (±SE) changes from randomization to the final follow-up visit. The values shown as “Chronic” represent the mean (±SE) changes from 2 months after the first dose of empagliflozin or placebo to the final follow-up visit. The mean changes in each trial group were estimated with the use of shared parameter models. For the plot, linear mixed models for repeated measures analyses were used to estimate the mean estimated GFR in each group at each scheduled follow-up visit (prespecified exploratory assessment). The vertical lines indicate the 95% confidence intervals for the estimated means. The coordinates of the boxes are shifted slightly on the x axis to avoid overlap.

# SUPPLEMENTARY TABLE 1: BASELINE CHARACTERISTICS BY PRE-SPECIFIED REGIONS

|  | **Europe** | | **North America** | | **China and Malaysia** | | **Japan** | |
| --- | --- | --- | --- | --- | --- | --- | --- | --- |
|  | Empagliflozin  (N=1344) | Placebo  (N=1304) | Empagliflozin  (N=844) | Placebo  (N=873) | Empagliflozin  (N=812) | Placebo  (N=820) | Empagliflozin  (N=304) | Placebo  (N=308) |
| **DEMOGRAPHICS** |  |  |  |  |  |  |  |  |
| **Age** (years) | 65.5 (14.2) | 65.4 (13.7) | 69.2 (10.8) | 68.6 (11.2) | 55.6 (13.1) | 55.0 (13.4) | 64.4 (12.2) | 66.2 (11.8) |
| **Female sex** | 390 (29.0) | 384 (29.4) | 336 (39.8) | 356 (40.8) | 285 (35.1) | 280 (34.1) | 86 (28.3) | 75 (24.4) |
| **Race** |  |  |  |  |  |  |  |  |
| White | 1259 (93.7) | 1238 (94.9) | 680 (80.6) | 682 (78.1) | 0 (0.0) | 0 (0.0) | 0 (0.0) | 0 (0.0) |
| Black | 22 (1.6) | 17 (1.3) | 106 (12.6) | 117 (13.4) | 0 (0.0) | 0 (0.0) | 0 (0.0) | 0 (0.0) |
| Asian | 43 (3.2) | 33 (2.5) | 37 (4.4) | 38 (4.4) | 810 (99.8) | 820 (100.0) | 304 (100.0) | 308 (100.0) |
| Mixed | 9 (0.7) | 3 (0.2) | 5 (0.6) | 4 (0.5) | 0 (0.0) | 0 (0.0) | 0 (0.0) | 0 (0.0) |
| Other | 11 (0.8) | 13 (1.0) | 16 (1.9) | 32 (3.7) | 2 (0.2) | 0 (0.0) | 0 (0.0) | 0 (0.0) |
| **PRIOR DISEASE** |  |  |  |  |  |  |  |  |
| **Diabetes** |  |  |  |  |  |  |  |  |
| Yes | 543 (40.4) | 508 (39.0) | 530 (62.8) | 536 (61.4) | 317 (39.0) | 316 (38.5) | 135 (44.4) | 155 (50.3) |
| No | 801 (59.6) | 796 (61.0) | 314 (37.2) | 337 (38.6) | 495 (61.0) | 504 (61.5) | 169 (55.6) | 153 (49.7) |
| **Diabetes type** |  |  |  |  |  |  |  |  |
| Type 1 | 18 (1.3) | 15 (1.2) | 16 (1.9) | 18 (2.1) | 0 (0.0) | 1 (0.1) | 0 (0.0) | 0 (0.0) |
| Type 2 | 520 (38.7) | 490 (37.6) | 513 (60.8) | 515 (59.0) | 310 (38.2) | 313 (38.2) | 127 (41.8) | 148 (48.1) |
| Other/unknown | 5 (0.4) | 3 (0.2) | 1 (0.1) | 3 (0.3) | 7 (0.9) | 2 (0.2) | 8 (2.6) | 7 (2.3) |
| **Cardiovascular disease** |  |  |  |  |  |  |  |  |
| Yes | 451 (33.6) | 443 (34.0) | 258 (30.6) | 285 (32.6) | 112 (13.8) | 126 (15.4) | 40 (13.2) | 50 (16.2) |
| No | 893 (66.4) | 861 (66.0) | 586 (69.4) | 588 (67.4) | 700 (86.2) | 694 (84.6) | 264 (86.8) | 258 (83.8) |
| **Primary kidney diagnosis** |  |  |  |  |  |  |  |  |
| Diabetic kidney disease | 295 (21.9) | 263 (20.2) | 391 (46.3) | 397 (45.5) | 252 (31.0) | 256 (31.2) | 94 (30.9) | 109 (35.4) |
| Hypertension/ renovascular | 310 (23.1) | 324 (24.8) | 219 (25.9) | 229 (26.2) | 136 (16.7) | 141 (17.2) | 41 (13.5) | 45 (14.6) |
| Glomerular | 357 (26.6) | 324 (24.8) | 77 (9.1) | 79 (9.0) | 317 (39.0) | 320 (39.0) | 102 (33.6) | 93 (30.2) |
| Other/unknown | 382 (28.4) | 393 (30.1) | 157 (18.6) | 168 (19.2) | 107 (13.2) | 103 (12.6) | 67 (22.0) | 61 (19.8) |
| **CLINICAL MEASUREMENTS** | |  |  |  |  |  |  |  |
| **Blood pressure (mmHg)** |  |  |  |  |  |  |  |  |
| Systolic | 136.7 (18.6) | 136.8 (18.3) | 133.8 (17.5) | 133.2 (17.8) | 139.5 (18.2) | 140.4 (18.8) | 133.9 (16.2) | 135.9 (17.3) |
| Diastolic | 78.2 (11.6) | 78.1 (11.4) | 73.8 (10.7) | 73.9 (11.2) | 82.4 (11.3) | 82.4 (11.8) | 77.4 (11.6) | 78.5 (12.5) |
| **Body mass index (kg/m^2^)** | 30.4 (6.2) | 30.5 (6.4) | 33.0 (7.5) | 33.2 (7.6) | 26.8 (5.2) | 26.8 (5.2) | 25.1 (4.1) | 25.3 (4.0) |
| **LABORATORY MEASUREMENTS** | |  |  |  |  |  |  |  |
| **Estimated GFR (mL/min/1.73m^2^)** |  |  |  |  |  |  |  |  |
| Mean (SD) | 34.8 (12.0) | 35.3 (12.3) | 35.3 (11.5) | 34.9 (10.9) | 40.5 (17.4) | 40.2 (17.5) | 46.2 (17.9) | 44.3 (18.4) |
| Distribution |  |  |  |  |  |  |  |  |
| <30 | 525 (39.1) | 501 (38.4) | 288 (34.1) | 314 (36.0) | 256 (31.5) | 264 (32.2) | 62 (20.4) | 72 (23.4) |
| ≥30 <45 | 617 (45.9) | 591 (45.3) | 433 (51.3) | 435 (49.8) | 315 (38.8) | 320 (39.0) | 102 (33.6) | 115 (37.3) |
| ≥45 | 202 (15.0) | 212 (16.3) | 123 (14.6) | 124 (14.2) | 241 (29.7) | 236 (28.8) | 140 (46.1) | 121 (39.3) |
| **Urinary albumin-to-creatinine ratio (mg/g)** |  |  |  |  |  |  |  |  |
| Geometric mean (95% CI) | 175  (157-195) | 167  (150-187) | 107  (93-123) | 121  (105-138) | 467  (418-521) | 493  (442-549) | 566  (484-661) | 593  (508-693) |
| Median (Q1-Q3) | 263  (32-931) | 218  (29-992) | 115  (19-577) | 125  (22-629) | 579  (213-1491) | 667  (232-1452) | 719  (293-1394) | 602  (293-1727) |
| Distribution |  |  |  |  |  |  |  |  |
| <30 | 321 (23.9) | 332 (25.5) | 265 (31.4) | 258 (29.6) | 63 (7.8) | 62 (7.6) | 16 (5.3) | 11 (3.6) |
| ≥30 <300 | 386 (28.7) | 386 (29.6) | 279 (33.1) | 289 (33.1) | 201 (24.8) | 191 (23.3) | 61 (20.1) | 71 (23.1) |
| ≥300 | 637 (47.4) | 586 (44.9) | 300 (35.5) | 326 (37.3) | 548 (67.5) | 567 (69.1) | 227 (74.7) | 226 (73.4) |
| **NTpro-BNP (ng/L)** | 209 (89-598) | 203 (92-525) | 213 (89-467) | 203 (80-537) | 105 (45-222) | 102 (43-232) | 88 (40-208) | 103 (45-215) |
| **MEDICATIONS** |  |  |  |  |  |  |  |  |
| **RAS inhibitor** | 1182 (87.9) | 1120 (85.9) | 681 (80.7) | 693 (79.4) | 707 (87.1) | 713 (87.0) | 261 (85.9) | 271 (88.0) |
| **Any diuretic therapy** | 696 (51.8) | 687 (52.7) | 444 (52.6) | 521 (59.7) | 161 (19.8) | 181 (22.1) | 61 (20.1) | 64 (20.8) |
| **Any lipid-lowering therapy** | 914 (68.0) | 903 (69.2) | 663 (78.6) | 689 (78.9) | 431 (53.1) | 403 (49.1) | 182 (59.9) | 193 (62.7) |
| Data are mean (SD) or median (Q1-Q3) for continuous data unless otherwise stated; and n (%) for categorical data. | | | | | | | | |

# SUPPLEMENTARY TABLE 2: EFFECT OF EMPAGLIFLOZIN ON PRIMARY AND SECONDARY OUTCOMES BY PRE-SPECIFIED REGIONS (POST-HOC EXPLORATORY ANALYSES)

|  | **Europe** | | **North America** | | **China and Malaysia** | | **Japan** | | **Overall** | | | Region P_het_ |
| --- | --- | --- | --- | --- | --- | --- | --- | --- | --- | --- | --- | --- |
|  | Empagliflozin  (N=1344) | Placebo  (N=1304) | Empagliflozin  (N=844) | Placebo  (N=873) | Empagliflozin  (N=812) | Placebo  (N=820) | Empagliflozin  (N=304) | Placebo  (N=308) | Empagliflozin  (N=3304) | Placebo  (N=3305) | Overall HR (95% CI) |  |
|  | n (%) | n (%) | n (%) | n (%) | n (%) | n (%) | n (%) | n (%) | n (%) | n (%) |  |  |
| **Primary outcome: progression of kidney disease or death from cardiovascular causes** | 188 (14.0) | 190 (14.6) | 87 (10.3) | 133 (15.2) | 124 (15.3) | 171 (20.9) | 33 (10.9) | 64 (20.8) | 432 (13.1) | 558 (16.9) | 0.72 (0.64-0.82) | 0.06 |
| **Key secondary outcomes** |  |  |  |  |  |  |  |  |  |  |  |  |
| Hospitalization for heart failure or death from cardiovascular causes | 68 (5.1) | 78 (6.0) | 39 (4.6) | 44 (5.0) | 18 (2.2) | 19 (2.3) | 6 (2.0) | 11 (3.6) | 131 (4.0) | 152 (4.6) | 0.84 (0.67-1.07) | 0.92 |
| Death from any cause | 70 (5.2) | 80 (6.1) | 50 (5.9) | 54 (6.2) | 21 (2.6) | 26 (3.2) | 7 (2.3) | 7 (2.3) | 148 (4.5) | 167 (5.1) | 0.87 (0.70-1.08) | 0.88 |
| **Other secondary outcomes** |  |  |  |  |  |  |  |  |  |  |  |  |
| Progression of kidney disease | 163 (12.1) | 165 (12.7) | 71 (8.4) | 111 (12.7) | 119 (14.7) | 165 (20.1) | 31 (10.2) | 63 (20.5) | 384 (11.6) | 504 (15.2) | 0.71 (0.62-0.81) | 0.06 |
| Death from cardiovascular causes | 30 (2.2) | 36 (2.8) | 20 (2.4) | 24 (2.7) | 6 (0.7) | 8 (1.0) | 3 (1.0) | 1 (0.3) | 59 (1.8) | 69 (2.1) | 0.84 (0.60-1.19) | 0.98 |
| End-stage kidney disease or death from cardiovascular causes^*^ | 75 (5.6) | 85 (6.5) | 41 (4.9) | 63 (7.2) | 37 (4.6) | 53 (6.5) | 10 (3.3) | 16 (5.2) | 163 (4.9) | 217 (6.6) | 0.73 (0.59-0.89) | 0.96 |
| The P_het_ is the p value for heterogeneity between treatment allocation and region. ^*^ End-stage kidney disease is defined as start of maintenance dialysis or receipt of a kidney transplant. | | | | | | | | | | | | |

# SUPPLEMENTARY TABLE 3: SENSITIVITY ANALYSIS FOR THE PRIMARY OUTCOME EXCLUDING 28 RANDOMIZED PARTICIPANTS FROM TWO SITES IN JAPAN

|  | **Empagliflozin**  **(N=3292)** | **Placebo**  **(N=3289)** |
| --- | --- | --- |
| Number (%) of events | 430 (13.1%) | 553 (16.8%) |
| Incidence rate per 100 years at risk (95% CI) | 6.85 (6.22-7.51) | 8.92 (8.19-9.68) |
| Hazard ratio vs. placebo (95% CI) | 0.73 (0.64-0.82) | |
| p-value | <0.0001 | |
| Cox model adjusted for age, sex, prior diabetes, eGFR category, urinary ACR category, and region. | | |

# COLLABORATORS

Recruiting collaborators from Japan included:

Chubu Rosai Hospital: Eitaro Nakashima, Rui Imamine, Makiko Minatoguchi, Yukari Miura, Miduki Nakaoka, Yoshiki Suzuki, Hitomi Yoshikawa; Shin Clinic: Koki Shin, Kanae Fujita, Misuzu Iwasa, Haruka Sasajima, Airi Sato; Kansai Electric Power Hospital: Yoshiyuki 8 Empagliflozin in Patients with Chronic Kidney Disease Hamamoto, Yuki Fujita, Takuya Haraguchi, Takanori Hyo, Kiyohiro Izumi, Toshiyuki Komiya, Sodai Kubota, Takeshi Kurose, Hitoshi Kuwata, Susumu Nakatani, Kaori Oishi, Saki Okamoto, Kaori Okamura, Jun Takeoka, Nagaaki Tanaka, Katsuya Tanigaki, Naohiro Toda, Koin Watanabe, Hiromi Komori, Rika Kumuji, Asako Takesada, Aya Tanaka; Nagoya University Hospital: Shoichi Maruyama, Tomonori Hasegawa, Akiko Ishiguro, Takuji Ishimoto, Kazuhiro Ito, Yutaka Kamimura, Noritoshi Kato, Sawako Kato, Hiroshi Kojima, Tomoki Kosugi, Kayaho Maeda, Masasi Mizuno, Shoji Saito, Hitomi Sato, Yuka Sato, Yasuhiro Suzuki, Akihito Tanaka, Yoshinari Yasuda, Fujiko Hasegawa, Maiko Hayashi, Shizuka Higashi, Kaho Shimamura, Momoko Sumi, Kazuki Tajima, Chimaki Unekawa, Kana Wakayama, Yukiko Wakita; Ota diabetes clinic: Takatoshi Otani, Ayako Imai, Sayaka Kawashima, Eri Kogure, Tomoe Sato, Misato Takezawa, Shinya Yoshida; Fukui Prefectural Hospital: Hideo Araki, Yuko Katsuda, Masahiro Konishi, Takahiro Matsunaga, Masashi Oe, Kunihiro Ogane, Masato Sakai, Tomoko Takahashi, Takahiro Yamano, Takuya Yokoyama, Hitomi Ito, Masayo Katayama, Emi Kuroda; Medical Corporation Seijinkai Ikeda Hospital: Toru Ikeda, Takuma Kojo, Etsuo Yoshidome, Rieko Mizumachi, Akane Yamamoto, Narihisa Yamasaki, Yoshihiko Yamasaki; Okayama University Hospital: Jun Wada, Jun Eguchi, Chigusa Higuchi, Akihiro Katayama, Masaru Kinomura, Masashi Kitagawa, Shinji Kitamura, Satoshi Miyamoto, Hiroshi Morinaga, Atsuko Nakatsuka, Ichiro Nojima, Kenichi Shikata, Hitoshi Sugiyama, Katsuyuki Tanabe, Kenji Tsuji, Haruhito Uchida, Mayu Watanabe, Chie Hashimoto, Takahiro Kato, Sayaka Yamamoto; Tokai University Hospital: Takehiko Wada, Masafumi Fukagawa, Naoto Hamano, Masahiro Koizumi, Hirotaka Komaba, Yosuke Nakagawa, Michiyo Iwamoto; Fukuoka University Hospital: Kosuke Masutani, Akane Katanosaka, Mayu Kiyota, Hikari Uchi, Yuka Ueda, Sonoka Yamamoto; Kawasaki Medical School Hospital: Hajime Nagasu, Seiji Itano, Tsukasa Iwakura, Hiroyuki Kadoya, Eiichiro Kanda, Naoki Kashihara, Kengo Kidokoro, Megumi Kondo, Tamaki Sasaki, Minoru Satoh, Atsuyuki Tokuyama, Reina Umeno, Yoshihisa Wada, Toshiya Yamamoto, Yu Yamanouchi, Masumi Abe, Yoko Inukai; Kobe University Hospital: Wataru Ogawa, Shunichiro Asahara, Hideki Fujii, Shunsuke Goto, Yushi Hirota, Tetsuya Hosooka, Keiji Kono, Shinichi Nishi, Yuko Okada, Kazuhiko Sakaguchi, Kenji Sugawara, Michiko Takahashi, Tomoko Takai, Yoshikazu Tamori, Kentaro Watanabe, Miyu Kitajima, Misaki Nishi, Junko Wada; Aichi Medical University Hospital: Yasuhiko Ito, Hideki Kamiya, Akimasa Asai, Nao Asai, Saeko Asano, Shogo Banno, Yohei Ejima, Hanako Hase, Tomohide Hayami, Tatsuhito Himeno, Takahiro Ishikawa, Mayumi Ito, Shiho Iwagaitsu, Rina Kasagi, Yoshiro Kato, Makoto Kato, Koichi Kato, Takayuki Katsuno, Miyuka Kawai, Hiroshi Kinashi, Masaki Kondo, Masako Koshino, Naoya Matsuoka, Yoshiaki Morishita, Mikio Motegi, Jiro Nakamura, Hiromi Shimoda, Hirokazu Sugiyama, Shin Tsunekawa, Makoto Yamaguchi, Kazuyo Takahashi; Juntendo University Hospital: Hirotaka Watada, Takashi Funayama, Yasuhiko Furukawa, Tomohito Gohda, Hiromasa Goto, Hideyoshi Kaga, Yasuhiko Kanaguchi, Akio Kanazawa, Kayo Kaneko, Toshiki Kano, Masao Kihara, Shogo Kimura, Takashi Kobayashi, Masayuki Maiguma, Yuko Makita, Satoshi Mano, Tomoya Mita, Takeshi Miyatsuka, Maki Murakoshi, Masahiro Muto, Masami Nakata, Junichiro Nakata, Yuya Nishida, Nao Nohara, Takeshi Ogihara, Daisuke Sato, Junko Sato, Hiroaki Sato, Yusuke Suzuki, Ruka Suzuki, Hitoshi Suzuki, Miyuki Takagi, Yoshifumi Tamura, Toyoyoshi Uchida, Seiji Ueda, Miki Asawa, Minako Miyaji, Eri Nagashima, Yoshie Shibata, Eri Yanagisawa; The University of Tokyo School of Medicine/Toranomon Hospital: Takashi Kadowaki, Toshimasa Yamauchi, Masaomi Nangaku, Yosuke Hirakawa, Hiroshi Nishi, Nobuhiro Shojima, Satoko Horikawa, Yukiko Nakayama, Naoko Yamada, Yuki Omori; Maebashi Hirosegawa Clinic: Shintaro Yano, Miyabi Ioka, Nahoko Kuwabara, Remi Nagano, Megumi Nozawa, Yumi Osawa; Shiga University of Medical Science Hospital: Hiroshi Maegawa, Shinji Kume, Shinichi Araki, Itsuko Miyazawa, Katsutaro Morino, Ikuko Kawai, Masumi Sobata, Motoko Takaoka; Koukan Clinic: Yasushi Iwaita, Takashi Udagawa, Ami Inamori, Aya Kawase, Aya Yamanaka; University of Tsukuba Hospital: Hitoshi Shimano, Akiko Fujita, Hitoshi Iwasaki, Hirayasu Kai, Yoshinori Osaki, Chie Saito, Motohiro Sekiya, Ryoya Tsunoda, Kunihiro Yamagata, Rikako Nakamura, Aiko Yamada; Center Hospital of the National Center for Global Health and Medicine: Mitsuru Ohsugi, Motoharu Awazawa, Ryotaro Bouchi, Shota Hashimoto, Makiko Hashimoto, Tomoko Hisatake, Noriko Ihana, Koko Ishizuka, Kazuo Izumi, Hiroshi Kajio, Michi Kobayashi, Noriko 9 Empagliflozin in Patients with Chronic Kidney Disease Kodani, Koji Maruyama, Michihiro Matsumoto, Maya Matsushita, Tomoka Nakamura, Takehiro Sugiyama, Akiyo Tanabe, Aiko Terakawa, Kojiro Ueki, Yuko Orimo, Takako Ozawa, Eriko Takahira; AMC Nishi-Umeda Clinic: Yoshimitsu Yamasaki, Masakazu Haneda, Tadahiro Tomita, Saori Akimoto, Akihiro Fujimoto, Kenji Ishihara, Chiho Murakami, Akiyo Nishiyama, Yukiko Toyonaga, Kana Uozumi, Yukihiro Yamaji; Jyoumou Ohashi Clinic: Tetsuya Shigehara, Jun Okajyo, Yukihiro Shimizu; Iwasaki internal medicine clinic: Shingo Iwasaki, Yuki Fukao, Megumi Furusho, Shintaro Nunokawa; Tohoku University Hospital: Hideki Katagiri, Tomohito Izumi, Keizo Kaneko, Shinjiro Kodama, Mariko Miyazaki, Yuichiro Munakata, Tasuku Nagasawa, Yuji Oe, Hiroto Sugawara, Kei Takahashi, Kazushige Hirata, Keiko Inomata, Shoko Otomo, Taeko Uchida, Chigusa Yamashita; Tokyo-eki Center-building Clinic: Arihiro Kiyosue, Ryota Tamura.
